# Is the Benefit of Treating Iron Deficiency Greater in Acute Heart Failure with Renal Dysfunction?

**DOI:** 10.3390/life13040915

**Published:** 2023-03-31

**Authors:** Raquel López-Vilella, Borja Guerrero Cervera, Víctor Donoso Trenado, Ignacio Sánchez-Lázaro, Luis Martínez Dolz, Luis Almenar Bonet

**Affiliations:** 1 Heart Failure and Transplantation Unit, Hospital Universitari i Politècnic La Fe,106, 46026 Valencia, Spain; 2 Cardiology Department, Hospital Universitari i Politècnic La Fe, 106, 46026 Valencia, Spain; 3 Centro de Investigación Biomédica en Red de Enfermedades Cardiovasculares (CIBERCV), Instituto de Salud Carlos III, 28029 Madrid, Spain

**Keywords:** heart failure, iron deficiency, renal dysfunction, cardio-renal syndrome, prognosis

## Abstract

Background: This study aims to analyse whether in acute heart failure (AHF) with iron deficiency (ID), the administration of ferric carboxymaltose (FCM) produces a greater benefit in renal dysfunction. Methods: A total of 812 consecutive patients admitted for AHF and ID were studied. Untreated (n:272) and treated (n:540) patients were compared. The six-month prevalence of a combined event (readmission for HF, all-cause death, and emergency department visit for decompensation) was analysed. Three grades of renal dysfunction (KDIGO) were compared, Group 1 (grades 1 and 2), Group 2 (grades 3a and 3b), and Group 3 (grades 4 and 5). Results: There were differences in sex distribution (untreated group: males 39.7% vs. treated group: males 51.9%; *p* < 0.001). Sex-adjusted combined event analysis showed a greater benefit in Group 1 (OR: 0.31, 95% CI:0.19–0.5; *p* < 0.001) and Group 2 (OR: 0.23, 95% CI:0.14–0.38; *p* < 0.001), but not in Group 3 (OR: 0.51, 95% CI:0.17–0.55; *p*: 0.237). Conclusions: The administration of FCM in patients with AHF and ID reduces the combined event analysed. The benefit is greater when renal dysfunction is present, except in very advanced degrees where no significant benefit is obtained.

## 1. Introduction

Iron deficiency (ID) is a very common comorbidity in patients with heart failure (HF) [1]. About half of patients with HF has ID [2,3,4], with specific prevalence estimates for ID in chronic HF ranging between 47 and 68% depending on the definition used for ID [5]. A slightly higher prevalence has been observed in general in preserved ejection fraction (HFpEF) vs. reduced ejection fraction (HFrEF) [6,7]. Insufficient dietary iron, reduced iron absorption due to the low-grade inflammation associated with congestion, and increased blood loss due to anti-thrombotic therapy or renal disease may all cause ID [8]. Several clinical trials have already demonstrated that ID is an important therapeutic target in patients with HF, especially in HFrEF. In chronic HF, administration of ferric carboxymaltose (FCM) results in short-term (30 days) incorporation of iron into myocytes with an improvement of the left and right ventricular function [9,10]. In the medium term (3 months), a study by our group analysed the response of patients with HF to intravenous iron administration according to the type of HF, finding that intravenous iron administration improves ejection fraction and cardiac functional status in outpatients with ID and HF with both preserved and reduced ejection fractions [11]. Another study in the same line of research that was conducted on 890 patients who were consecutively admitted for acute heart failure (AHF) and received FCM administration before discharge showed a reduction in the combined event at 6 months (overall mortality, emergency department visits for decompensation, and readmissions) with a NNT close to 10 [12]. Anaemia and chronic kidney disease are independent predictors of mortality and excess hospitalisations in patients with HF with or without a reduced left ventricular systolic function [13]. On the other hand, the presence of renal dysfunction in these patients (cardiorenal syndrome) has been estimated to be up to 70% [14,15]. There are studies suggesting that the beneficial effect of iron administration may be different depending on different degrees of renal impairment, such that patients with greater renal dysfunction would benefit more from iron administration [13,16]. This work hypothesised that, as the glomerular filtration rate (GFR) decreases, the beneficial effect of iron administration in iron-deficient patients would be greater in terms of morbidity and mortality.

Thus, the study aimed to analyse, in a large series of patients admitted for acute HF and ID, whether the benefit of administering FCM vs. not administering it is greater in cases with worse renal function.

## 2. Materials and Methods

**Figure 1:**
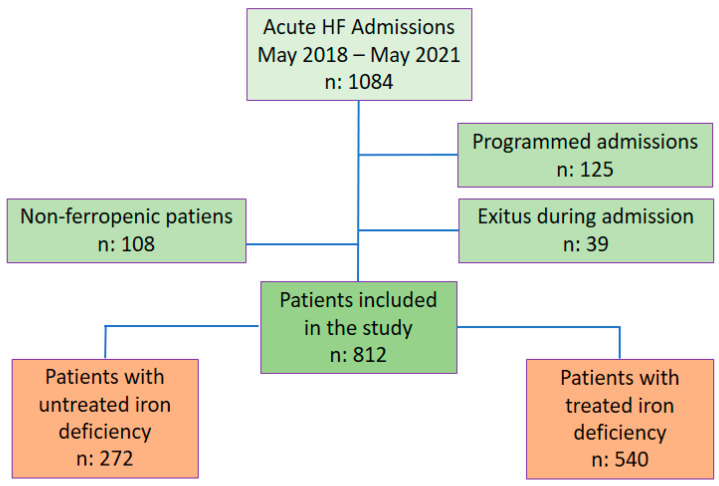
Flow chart. Abbreviations: HF: Heart failure.

A total of 1084 patients consecutively admitted with a diagnosis of acute HF in any of its forms (acute pulmonary oedema, systemic congestion, mixed (pulmonary and systemic) congestion and low cardiac output) to the Cardiology Department of a referral hospital were recruited. Patients transferred from other clinical services, those who died during admission, and those who did not exhibit ID according to the criteria of the European HF guidelines [16] were excluded. A total of 812 patients were included in the retrospective analysis over 3 years (May 2018–May 2021), and the follow-up was within 6 months (Figure 1).

Patients were divided into 2 groups depending on the presence of ID and treatment: (a) treated iron deficiency or (b) untreated iron deficiency. In all cases, the iron compound used was FCM. The decision whether to treat the patient during admission was made by the responsible physician. The groups were further divided, according to renal function quantified by glomerular filtration rate (GFR), into three groups: normal dysfunction (GFR > 60 mL/min/1.73 m^2^), moderate dysfunction (GFR 30–60 mL/min/1.73 m^2^), and severe dysfunction GFR < 30 (mL/min/1.73 m^2^). Clinical, analytical, echocardiographic, and treatment variables were analysed in each study group. After hospital discharge and during the follow-up period, the number of visits to the hospital emergency department, the number of readmissions, and mortality rates were recorded. A period of 6 months is the usual follow-up time for all patients admitted for decompensated HF and was chosen as the endpoint for the study.

All patients underwent a preconfigured analytical profile the day after hospitalisation, which included ferric parameters (ferritin levels and transferrin saturation index (TSAT)). ID was diagnosed and treated with FCM according to the criteria established in the European HF guidelines (ferritin levels < 100 µg/L or 100–300 µg/L accompanied by a TSAT < 20%) [16]. The dose of FCM administered was 1000 mg diluted in 250 cc of 0.9% saline solution, infused over 30 min, or the same dose diluted in 100 cc and infused over 15 min. For patients weighing < 50 kg, 500 mg was administered in the same diluent and for the same time. For patients with haemoglobin levels ≥ 14 g/dL, the administered dose of CMF was 500 mg.

This study was conducted following the Declaration of Helsinki and was approved by the Biomedical Research Ethics Committee of the Hospital Universitario y Politécnico La Fe, Valencia (Spain).

### Statistical Analysis

To describe the sample characteristics, we used the absolute (n) and relative frequency (%) for qualitative variables, and mean (standard deviation) for the quantitative variables if normal (Kolmogorov–Smirnov test) or otherwise median (25th and 75th percentiles). Then, a comparison between the treatment groups was performed by Pearson chi-square test (Fisher test if small samples were used, and two categories or the maximum likelihood correction if more than two groups) for qualitative variables; for quantitative variables, Student’s T test was used for independent samples if normal and the Mann–Whitney U test was used if otherwise. Next, chronic kidney disease (CKD) was classified into three groups: CKD stages 1/2, 3a/3b, and 4/5. Significance of the differences between the renal dysfunction groups were compared using Pearson’s χ^2^, chi-square test, and maximum likelihood ratio, if necessary. To estimate the potential benefit of treatment, we calculated the absolute risk reduction (ARR) as the difference between the percentage of the outcome in each treatment group (treated and untreated), and the relative risk reduction (RRR) as ARR divided by the percentage in the untreated group. The number of patients needed to treat to avoid one event (NNT) was calculated as the inverse of the ARR. These results were obtained for each CKD group. Lastly, sex-adjusted odds ratios (OR) of each outcome associated with treatment group, and their corresponding 95% confidence intervals, were obtained using binary logistic regression, and forest plots were added. Two-tail *p* value of 0.05 was considered as statistical significance. All analyses were performed using SPSS v.28.0 software (IBM Corp. Released 2021. IBM SPSS Statistics for Windows, Version 28.0. Armonk, NY, USA: IBM Corp).

## 3. Results

### 3.1. Baseline Characteristics

The mean age of the included patients was 75 years. The underlying heart diseases were mainly ischaemic, hypertensive, and valvular heart disease. The most frequent comorbidities were dyslipidaemia and atrial fibrillation (AF). About one-third of the patients had a history of renal failure. In most cases, HF was already diagnosed before admission (non-de novo HF). Baseline functional status before admission (NYHA) was between II and III/IV. The most frequent reason for decompensation was disease progression without an identifiable cause. The most frequent haemodynamic pattern on admission was pulmonary congestion. In most cases, HF was HFpEF with normal right ventricular function. Advanced degrees of renal dysfunction (G 4 and 5) were uncommon. The mean glomerular filtration rate was below 60 mL/min/1.73m^2^ with NT-ProBNP above 5000 pg/mL. Ferritin values were between 100 and 300 ng/mL with TSAT < 20%. More than half of the patients were treated with anticoagulants and were taking loop diuretics, renin-angiotensin-aldosterone system inhibitors (RAASi), and beta-blockers (Table 1 and Table 2). As can be appreciated from Table 2, there were no differences observed in the treatment of heart failure at hospital discharge, so it is not expected that the optimisation of medical therapy would have influenced the results.

### 3.2. Comparison between Groups

Few statistically significant differences were found when comparing history, baseline clinical, and echocardiographic characteristics between the two groups. The most relevant were the unequal distribution of sex (*p* < 0.001) and some degrees of renal function (*p* < 0.05). No significant differences were found concerning the admission blood tests. However, some variables were close to significance (*p*: 0.1–0.05) such as creatinine, bilirubin, AST, and LDL-cholesterol. The drugs prescribed at discharge showed no differences between the two groups. All these characteristics can be seen in Table 1 and Table 2.

### 3.3. Event and Combined Risk

The results are presented in three renal dysfunction groups (Table 3). In the analysis of events by treatment group and renal function, it was observed that there are differences in the response to iron depending on the degree of renal dysfunction. When adjusted for sex, some events lost significance, such as readmission and exitus for the most severe dysfunctions (G 4 and 5). The combined event showed that when comparing treated vs. untreated patients, there is a greater benefit after iron administration in groups G 3a and 3b than in groups G 1 and 2. It could be seen that the groups with very advanced renal dysfunction (G 4 and 5) do not benefit from iron administration.

**Figure 2:**
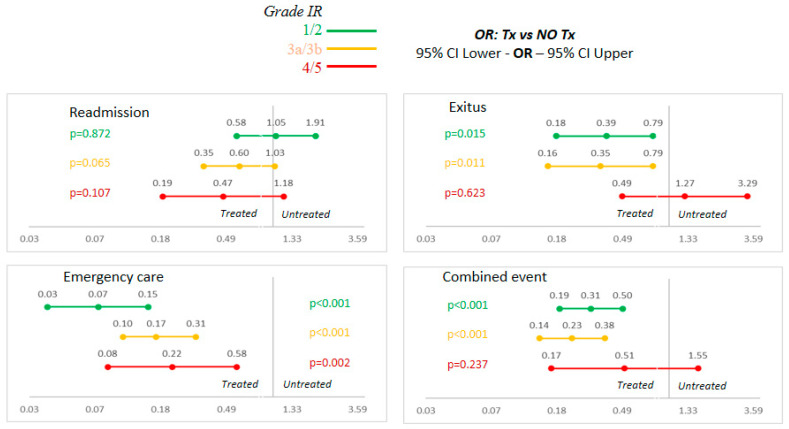
Sex-adjusted multivariate analysis. Abbreviations: FR: renal failure; NO Tx: untreated; OR: odds ratio; Tx: treated.

The chart shows the benefit of administering iron by study group in the multivariate analysis adjusted for sex. The overall tendency is to improve the benefit of treatment the worse the renal function is. This is mainly at the expense of the “exitus” and “emergency case” groups. For the combined event, treating ID resulted in a 69% risk reduction in groups 1 and 2 (OR: 0.31, 95% CI: 0.19–0.5, *p* < 0.001) and an even greater reduction of 77% in groups 3a and 3b (OR: 0.23, 95% CI: 0.14–0.38, *p* < 0.001). However, in patients with very impaired renal function (G 4 and 5) there was also a risk in reduction, which, in this case, statistical significance was not reached (OR: 0.51, 95% CI: 0.17–1.55, *p*: 0.237). The adjusted results can be seen in Figure 2.

## 4. Discussion

ID is a very common comorbidity in acute and chronic HF. The aetiology of ID in worsening HF is complex and multifactorial and seems to consist a combination of reduced iron uptake (malnutrition and fluid overload), impaired iron storage (inflammation and chronic kidney disease), and iron loss (anti-platelets) [17,18,19]. Iron administration in these patients improves functional status, cardiac function, and hospitalisation [20,21,22]. Based on this prognostic relevance, the 2021 European Society of Cardiology guidelines on HF provide a Class IIa, level of evidence A, recommendation for FCM use in symptomatic HF patients with ejection fraction < 45%, and a Class IIa, level of evidence B, recommendation for FCM in patients with HF recently hospitalised for HF and ejection fraction < 50% to reduce the risk of HF hospitalisation [16]. In the recent past, it has been suggested in the medical literature that the benefit of treating ID may be greater in patients with a worse renal function [13,16]. Previous studies by our clinical research group have shown that, in patients with acute HF and iron deficiency, the administration of FCM reduces the combined event of emergency care, all-cause mortality, and re-hospitalisation at 6 months [12]. In the current sub-study, a sub-analysis was performed with the same patients to determine whether the effect of iron repletion is greater in the presence of renal dysfunction. It has been observed that, indeed, the benefit of treating iron-deficient patients is greater in advanced degrees of renal dysfunction. However, in more extreme degrees (G 4 and 5, GFR < 30 mL/min/1.73 m^2^), this benefit is lost.

The number of patients included in the analysis was substantial, exceeding the 304 patients in the CONFIRM-HF [21] and with a similar number to 1108 in the AFFIRM-AHF [20], making it a representative sample in this field of scientific interest. The patients analysed had baseline characteristics similar to the groups studied in the main trials on intravenous ferrotherapy in HF, with an age slightly above 70 years and multiple comorbidities, including chronic kidney disease (predominantly KDIGO stages 2 and 3) [12,20,22,23]. The most frequent baseline functional class II/IV (55%) was similar to the CONFIRM clinical trial (53%), with a slight predominance in males [21]. On admission, the predominant haemodynamic pattern in both groups was pulmonary congestion, which is the main cause of urgent medical attention and hospital admission in patients with HF, isolated or together with systemic congestion [23,24]. This decompensation profile is especially frequent in the HFpEF group with normal right ventricular function; this type of HF was frequent in both study groups [24,25]. Regarding the renal function of the patients analysed, the predominant KDIGO stage in CKD patients was stage 2, followed by stage 3, with advanced grades (stages 4 and 5) being rare in the sample. In patients with CKD, ID and anaemia are frequent complications that increase as glomerular filtration rate decreases [26]. This is probably why in the majority of studies of ferrotherapy and HF, the predominant stages are 2 and 3 [20,22,27], as patients in more advanced stages are usually in a situation of frank anaemia, usually suffer from a deficiency in the production of erythropoietin, and require more intensive treatment and specific management [26,28]. Thus, patients with more advanced stages of renal failure benefit less from the administration of iron in any of its forms in relation to the erythropoietin deficit, not necessarily in relation to the heart failure condition. Nevertheless, there are no studies evaluating these aspects in the setting of acute heart failure.

Laboratory tests on admission showed some differences between the two groups. Some parameters were close to significance, such as bilirubin and AST, but differences were considered to be of no clinical relevance. The definition of ID and the cut-offs have been borrowed from the field of nephrology, where they were suggested to have good performance in terms of sensitivity and specificity for the diagnosis of ID, but also as targets for iron therapy [8,29].

The main difference found between the two groups was the unequal sex distribution, with a predominance in women of the untreated group. Similar to most HF pharmacological studies, there is a higher proportion of female patients not treated for various reasons [23,30]. For this reason, a sex-adjusted multivariate analysis was carried out to correct the results of that variable.

The primary endpoint was defined as a combined event that included readmissions for HF, all-cause exitus, and the urgent consultation for decompensation. It was decided to include unscheduled visits for decompensation in the endpoint because, with the increasing development in HF units and cardiorenal units, non-severe decompensation can often be managed on an outpatient basis by the administration of intravenous diuretics, without requiring hospital admission [15,24,25,26].

In the analysis of events by treatment group and renal function, it was observed that there are differences in the response to iron depending on the degree of renal dysfunction. Thus, the combined event showed a greater benefit after iron administration in groups G 3a and 3b than in groups G 1 and 2. The clinical benefit of correcting iron deficiency in acute HF is well known; however, it has not been proven to date that the benefit is greater in patients with a more severe degree of renal dysfunction. This information was suggested in the form of theories or hypotheses in previous studies; however, it had not been objectified in actual clinical practice. The mechanisms underlying these differences are probably multifactorial. Iron is an essential micronutrient, and the repletion of iron stores has positive effects on all tissues, from increasing the contractile function of cardiomyocytes [9,15] to the proper functioning of renal nephron cells, conferring a certain renal protective effect [31]. On the other hand, HF produces a proinflammatory state [18]. The liver under inflammation stimulates the synthesis of hepcidin, which blocks iron absorption leading to increased sequestration in the enterocyte [28]. As renal dysfunction increases, the elimination of proteins, immunoglobulins, and other inflammatory molecules decreases, thereby increasing the synthesis of hepcidin [29]. Therefore, ID observed with cardiorenal diseases may be due to the increased levels of hepcidin linked with a chronic inflammatory status, which leads to decreased iron absorption and mobilisation from the reticuloendothelial system [8]. Although the pathophysiology of iron in the context of HF and CKD is complex, this mechanism, together with others, could justify the findings of a greater benefit in the presence of worse renal function, since we provide bioavailable iron to patients who have a greater tendency to intracellular iron sequestration. In severe HF, especially if renal failure is associated, beyond the inflammation, a contributing cause of ID might be the reduction in iron absorption in the bowel due to an HF-related generalised oedema [32,33,34]. Interestingly, this study has shown that in groups with very advanced renal dysfunction (G 4 and 5), the benefit of correcting iron deficiency is lost. The different responses to treatment according to the degree of renal dysfunction are maintained in the multivariate analysis adjusted for sex, confirming that in groups 3a and 3b, it is necessary to treat only 3.1 patients to obtain the benefit of the combined event [31,32]. In the more advanced stages (G 4 and 5), readmission and exitus events lost statistical significance, as did the combined event, so we cannot affirm that they benefit from the administration of iron. However, there was a reduction in risk which, although not statistically significant, was clinically important, and emergency department visits were reduced. These patients in more advanced stages present so many systemic complications due to their renal pathology (sarcopenia, osteodystrophy, severe anaemia, calciphylaxis, hyperparathyroidism, etc.) that the beneficial effect of the treatment is probably diluted by the magnitude and prognostic implication of these conditions [35]. However, at present, the KDIGO (Kidney Disease: Improving Global Outcomes) guidelines on anaemia management in CKD suggest the treatment of anaemia with iron supplementation in adults with CKD in stages 3–5, when there is ID. The goal is to balance transfusion avoidance with the risks and side effects of iron therapy [36].

These results highlight the importance of always determining iron metabolism in HF patients with or without renal disease. For this purpose, it is helpful to have pre-configured analytical profiles that include iron metabolism both in the clinic and hospital ward [37,38], as well as in the development of strong cardio-renal units [39], to prevent HF patients from reaching advanced stages of renal failure, where the benefit of treatment could be smaller. The final aim is to try to offer our patients the best treatment of comorbidities in less advanced stages of cardiorenal syndrome.

### Study Limitations

The main limitation of the study is its retrospective nature, even though the groups were homogeneous. As this is a retrospective study, it is possible that there may be other variables that have not been taken into account in the analysis, especially with regard to renal failure. Thus, with regard to renal function, we have taken into account the creatinine value and the glomerular filtration rate without evaluating other factors, such as cystatin C, microalbuminuria, etc.

At the same time, the data from the daily clinical practice provide very relevant information as they reflect the reality of patient management. In addition, the number of patients included is large and the subgroups are generally well-balanced. The study was carried out in a single hospital centre, so that data entry coincides with admission and was always carried out by cardiologists with expertise in HF, thus minimising errors. A follow-up of 6 months was chosen as this is the standard time criterion for monitoring patients admitted for decompensated HF, but it may be insufficient time to assess the effects on cardiovascular or HF mortality.

Despite these limitations, this study is the first to analyse—in real life and with a large number of patients—the effectiveness of FCM in subjects with decompensated AHF, both reduced and preserved, and its effectiveness on the different degrees of renal dysfunction. No other studies have been found in the literature comparing the effects of intravenous ferrotherapy by kidney disease groups.

Although prospective studies will be necessary, given the lack of articles analysing the correction of iron deficiency in acute heart failure independent of left ventricular ejection fraction and according to the degree of renal failure, this information may be useful for clinicians who manage this complex pathology on a daily routine basis.

Given the results obtained, it can be recommended to administer FCM to all patients admitted for AHF with ID to prevent readmissions, cardiac decompensation, and overall mortality. Although in more advanced stages of renal dysfunction, the benefit seems to be less, it is recommended to administer it, as it is a safe drug and may be beneficial on other variables not analysed or in the longer term.

## 5. Conclusions

The administration of FCM to patients admitted for acute HF with ID reduces the combined six-month event of overall mortality, rehospitalisation for HF, and the need to visit the emergency department for decompensation. The benefit is greater in patients with greater renal dysfunction except in very advanced degrees where no significant benefit is obtained.

## Figures and Tables

**Table 1:** Baseline characteristics.

	NOT Treated	Treated	
	n = 272	n = 540	*p*
Antecedents (n, %)			
Age (years) (*)	75.8 ± 11.4	75.9 ± 10.5	0.131
Male	108 (39.7)	280 (51.9)	0.001
Baseline heart disease (n, %)			
IHD	57 (21.0)	124 (23.0)	0.576
VHD	84 (30.9)	162 (30.0)	0.859
AF	27 (9.9)	43 (8.0)	0.419
DCM	30 (11.0)	64 (11.9)	0.818
HT	57 (21.0)	136 (25.2)	0.212
Other	17 (6.3)	11 (2.0)	0.004
History (n, %)			
CVS	44 (16.2)	102 (18.9)	0.394
HT	237 (87.1)	458 (84.8)	0.434
Dyslipidaemia	158 (58.1)	318 (58.9)	0.886
DM	157 (57.7)	331 (61.3)	0.365
Smoking	35 (12.9)	51 (9.4)	0.169
Alcoholism	16 (5.9)	28 (5.2)	0.803
COPD	49 (18.0)	113 (20.9)	0.375
SAHS	38 (14.0)	87 (16.1)	0.487
Obesity (BMI > 30 kg/m^2^)	66 (24.3)	114 (21.1)	0.352
Renal failure	84 (30.9)	138 (25.6)	0.128
Hypothyroidism	26 (9.6)	50 (9.3)	0.991
AF	103 (37.9)	178 (33.0)	0.191
Stroke	30 (11.0)	62 (11.5)	0.941
PVD	12 (4.4)	37 (6.9)	0.222
Clinical characteristics (n, %)			
Nº previous admissions (*)	0.8 ± 0.7	0.9 ± 0.7	0.052
HF de novo	106 (39.0)	182 (33.7)	0.161
FS (NYHA)			
I	46 (16.9)	77 (14.3)	0.373
II	150 (55.1)	288 (53.3)	0.678
III	63 (23.2)	144 (26.7)	0.319
IV	13 (4.8)	30 (5.6)	0.764
Cause decompensation			
Arrhythmia	56 (20.6)	118 (21.9)	0.746
Infectious	17 (6.3)	42 (7.8)	0.517
Ischemic	23 (8.5)	30 (5.6)	0.153
Disease progression	110 (40.4)	199 (36.9)	0.359
Unknown	34 (12.5)	64 (11.9)	0.878
HT	36 (13.2)	68 (12.6)	0.883
Hemodynamic pattern			
Pulmonary congestion	188 (69.1)	363 (67.2)	0.641
Systemic + pulmonary congestion	39 (14.3)	54 (10.0)	0.086
Systemic congestion	45 (16.6)	84 (15.6)	0.793
Low cardiac output	0 (0.0)	8 (1.5)	0.101
Echocardiography (n, %)			
LVEF (*)	45.0 ± 9.6	46.0 ± 10.2	0.176
LVEF preserved (≥50%)	190 (69.9)	351 (65.0)	0.192
RV function			
Normal	198 (73.0)	402 (74.4)	0.674
Mild depression	38 (14.5)	74 (13.7)	0.997
Moderate depression	32 (11.8)	49 (9.1)	0.279
Severe depression	4 (1.4)	15 (2.8)	0.359
Renal function (KDIGO 2012)			
G 1 (≥90 mL/min/1.73 m^2^)	25 (9.2)	59 (10.9)	0.444
G 2 (60–89 mL/min/1.73 m^2^)	102 (37.5)	164 (30.4)	0.04
G 3a (45–59 mL/min/1.73 m^2^)	62 (22.8)	86 (15.9)	0.017
G 3b (30–44 mL/min/1.73 m^2^)	55 (20.2)	130 (24.1)	0.217
G 4 (15–29 mL/min/1.73 m^2^)	18 (6.6)	84 (15.6)	0.001
G 5 (<15 mL/min/1.73 m^2^)	10 (3.7)	17 (3.1)	0.692
Renal function (study groups)			
G 1 y 2 (≥60 mL/min/1.73 m^2^)	127 (46.7)	223 (41.3)	0.143
G 3a/3b (30–59 mL/min/1.73 m^2^)	117 (43.0)	216 (40.0)	0.14
G 4 y 5 (≤29 mL/min/1.73 m^2^)	28 (10.3)	101 (18.7)	0.002

Data are expressed as % of number of cases and mean ± SD for normally distributed variables (*). *p* < 0.05 was considered statistically significant. Abbreviations: AF: atrial fibrillation; BMI: body mass index; COPD: chronic obstructive pulmonary disease; CVS: cardiovascular surgery; DCM: dilated cardiomyopathy; DM: diabetes mellitus; FS: functional status; HF: heart failure; HT: hypertension; IHD: ischaemic heart disease; LVEF: left ventricular ejection fraction; NYHA: New York Heart Association functional classification of the HF; PVD: peripheral vascular disease; RV: right ventricle; SAHS: sleep apnoea-hypopnea syndrome; SD: standard deviation; VHD: valvular heart disease.

**Table 2:** Analytical and pharmacological characteristics.

	NOT Treated	Treated	
	n = 272	n = 540	*p*
Admission analytics (#)			
Urea (mg/dL)	50.0 (46.0)	56.0 (55.0)	0.118
Creatinine (mg/dL) (*)	1.28 ± 0.77	1.39 ± 0.78	0.054
GFR (mL/min/1.73 m^2^) (*)	57.9 ± 23.9	55.0 ± 25.1	0.11
Bilirubin (mg/dL)	1.0 (0.7)	1.1 (0.7)	0.052
AST (U/L)	23.0 (16.0)	21.0 (14.0)	0.064
ALT (U/L)	22.6 (18.8)	23.0 (17.3)	0.243
TnT(u) (ng/mL)	45.9 (28.3)	44.0 (36.7)	0.461
NT-ProBNP (pg/mL)	5762 (3870)	5398 (3207)	0.151
Sodium (mEq/L)	141.5 (7.2)	142.0 (7.3)	0.358
Potassium (mEq/L)	4.1 (1.3)	4.2 (1.4)	0.327
Haemoglobin (g/dL)	13.5 (5.1)	13.2 (4.9)	0.422
Haematocrit (%)	40.6 (7.8)	40.7 (11.9)	0.132
Uric acid (mg/dL)	8.4 (4.7)	8.2 (4.4)	0.442
Cholesterol-HDL (mg/dL)	42.1 (23.3)	43.0 (16.3)	0.47
Cholesterol-LDL (mg/dL)	79.2 (24.7)	74.0 (42.6)	0.06
Triglycerides (mg/dL)	133.5 (84.1)	138.7 (72.0)	0.363
Ferritin (ng/mL)	130.1 (95.2)	126.0 (88.1)	0.45
TSAT (%)	16.3 (8.0)	15.5 (7.9)	0.172
HbA1c (%)	6.3 (0.8)	6.4 (0.9)	0.117
CA125 (U/mL)	73.0 (62.9)	70.9 (65.6)	0.334
Discharge treatment (n, %)			
IECAs/ARAII inhibitors	165 (60.7)	349 (64.6)	0.303
Beta-blockers	175 (64.3)	339 (62.8)	0.72
ARNI	42 (15.4)	70 (13.0)	0.39
MRA	105 (38.6)	190 (35.2)	0.38
SGLT2i	91 (33.5)	185 (34.3)	0.881
Ivabradine	21 (7.7)	43 (8.0)	0.986
Digoxin	70 (25.7)	131 (24.3)	0.709
Loop diuretics	256 (94.1)	506 (93.7)	0.939
Thiazides	52 (19.1)	97 (18.0)	0.76
Acetazolamide	4 (1.5)	10 (1.9)	0.914
Tolvaptan	16 (5.9)	29 (5.4)	0.89
Potassium supplement	33 (12.1)	59 (10.9)	0.693
Hypokalemic therapy	16 (5.9)	26 (4.8)	0.631
Antiplatelet agents	73 (26.8)	151 (28.0)	0.799
Anticoagulants	162 (59.6)	346 (64.1)	0.239
OAD (No iSGLT2)	102 (37.5)	195 (36.1)	0.756
Nitrates	29 (10.7)	65 (12.0)	0.644
Antiarrhythmic	57 (21.0)	104 (19.3)	0.632
Statins	157 (57.7)	286 (53.0)	0.226
Calcium antagonists	82 (30.1)	151 (28.0)	0.571
Pulmonary vasodilator	5 (1.8)	11 (2.0)	0.94
Alopurinol	65 (23.9)	124 (23.0)	0.834

Data are expressed as % of number of cases, mean ± SD for normally distributed variables (*) and median ± interquartile range for non-normally distributed variables (#). *p* < 0.05 was considered statistically significant. Abbreviations: ACE/ARA-II inhibitors: angiotensin-converting enzyme inhibitors/angiotensin II receptor antagonists; ARNI: angiotensin receptor-neprilysin inhibitors; AST: aspartate aminotransferase; CA125: cancer antigen 125; GFR: glomerular filtration rate; GOT: GPT: glutamic-pyruvic transaminase; MRA: mineralocorticoid receptor antagonists; NT-ProBNP: N-terminal pro-brain natriuretic peptide; TNT: troponin cardiac T; TSAT: transferrin saturation; HbA1c: glycated haemoglobin A1c; iSGLT2: sodium-glucose co-transporter inhibitors type 2; OAD: oral antidiabetic.

**Table 3:** Analysis of events by treatment group and by degree of renal failure.

	Events in Treated Patients. N = 540 (n,%)	Events in Patients NOT Treated. N = 272 (n,%)	Percentage Reduction (ARR) NOT tx-tx (95% CI)	*p*	RRR/NNT	Adjusted * (OR) (95% CI)	*p*
Readmission Event							
1/2	37 (16.6)	20 (15.7)	0.9 (2.5/1.5)	0.837	5.7/11.1	1.050 (0.578/1.908)	0.872
3a/3b	41 (19.0)	32 (27.4)	8.4 (3.4/13.4)	0.021	30.7/11.9	0.602 (0.352/1.031)	0.065
4/5	33 (32.7)	16 (57.1)	24.4 (8.5/40.3)	0.018	42.7/4.10	0.471 (0.189/1.175)	0.107
Exitus Event							
1/2	13 (5.8)	17 (13.4)	7.6 (3.0/12.2)	0.015	56.7/13.2	0.387 (0.180/0.785)	0.015
3a/3b	12 (5.6)	16 (13.7)	8.1 (3.4/13.4)	0.011	59.1/12.3	0.353 (0.159/0.785)	0.011
4/5	39 (38.6)	9 (32.1)	6.5 (15.6/5.3)	0.531	20.2/15.4	1.269 (0.490/3.285)	0.623
Emergency Department Visits Event							
1/2	10 (4.5)	46 (36.2)	31.7 (23.6/39.8)	<0.001	87.6/3.2	0.071 (0.033/0.151)	<0.001
3a/3b	32 (14.5)	53 (45.3)	30.5 (22.2/38.8)	<0.001	67.3/3.3	0.171 (0.103/0.311)	<0.001
4/5	15 (14.9)	10 (53.6)	38.7 (20.7/56.7)	<0.001	72.2/2.6	0.217 (0.082/0.577)	0.002
Combined Event							
1/2	60 (26.9)	66 (52.0)	25.1 (17.6/32.6)	<0.001	48.3/4.0	0.306 (0.190/0.495)	<0.001
3a/3b	69 (31.9)	75 (64.1)	32.2 (23.7/40.7)	<0.001	50.2/3.1	0.230 (0.140/0.377)	<0.001
4/5	66 (65.3)	53 (82.1)	16.8 (3.0/32.8)	0.089	20.5/6.0	0.510 (0.168/1.554)	0.237

* Adjustment by sex. ARR = Absolute risk reduction NNT = Number needed to treat. RRR = Relative risk reduction (NoTx-Tx/NoTx) × 100 Tx: treated. Abbreviations: NOT Tx: not treated; Tx: treated.

## Data Availability

Not applicable.
